# Arterial supply to the bleeding diverticulum in the ascending duodenum treated by transcatheter arterial embolization— a duodenal artery branched from the inferior pancreaticoduodenal artery

**DOI:** 10.1186/2193-1801-3-17

**Published:** 2014-01-09

**Authors:** Hiroki H Sanda, Nobuyuki N Kawai, Morio M Sato, Fumihiro F Tanaka, Kouhei K Nakata, Hiroki H Minamiguchi, Motoki M Nakai, Tetsuo T Sonomura

**Affiliations:** Department of Radiology, Wakayama Medical University, 811-1 Kimiidera, Wakayamashi, Wakayama, 641-8510 Japan

**Keywords:** Arterial bleeding, Diverticulum, Ascending duodenum, Transcatheter arterial embolization, n-butyl-cyano-acrylate

## Abstract

We present a case of endoscopically unmanageable hemorrhagic diverticulum in the ascending duodenum. The ventral and dorsal walls of the ascending duodenum were supplied from the first jejunal artery (1JA) and inferior pancreaticoduodenal artery (IPDA), respectively. The hemorrhage mainly occurred from IPDA. The abruptly branching of IPDA from superior mesenteric artery enabled successful catheterization of the IPDA with an angled microcatheter. Hemostasis was obtained by embolization using n-butyl cyanoacrylate. Gastroendoscopy depicted a duodenal hemi-circumferential ulcer. No symptoms related to hemorrhage were found at the last follow-up at 12 months.

## Introduction

The duodenum is comprised of four portions: the duodenal bulb, the descending duodenum, the transverse duodenum, and the ascending duodenum (Federle et al. 2010). Duodenal diverticulum is observed with an incidence of 20%–22% (Scundore et al. 1982; Dumonceau et al. 1998). Sakurai et al. accumulated 70 cases of hemorrhagic duodenal diverticulum; the site was the descending diverticulum in 40 cases, transverse duodenum in 22, ascending duodenum in 2, and undocumented in 6. Treatment was as follows: surgical diverticulectomy in 40 cases, endoscopic hemostasis in 20, transcatheter arterial embolization (TAE) in 3, and vasopressin infusion or watchful waiting in 3 (Sakurai et al. 2000). There are three reports of life-threatening hemorrhagic diverticulum in the ascending duodenum that were treated by surgical diverticulectomy (Balkissoon et al. 1992; Rioux et al. 1996; Yin et al. 2001)

We present a case of endoscopically unmanageable hemorrhage from a diverticulum in the ascending duodenum, which was treated by embolization of the first jejunal artery (1JA) and the inferior pancreaticoduodenal artery (IPDA).

## Case report

Institutional Review Board approval was not required for this case report. A 70-year-old woman presented to the Emergency Department of our hospital for evaluation of abdominal pain and tarry stool. She had been taking Plavix (clopidogrel hydrogen sulfate) for 3 years since a brain infarction. Laboratory tests revealed anemia (hemoglobin 7.6 g/dl). Immediately before gastro-endoscopy, the patient went into a state of shock, with blood pressure of 60 mmHg/0 and heart rate of 110 beats/minute. Tracheal intubation was performed and continuous infusion of venous Inovan was initiated.

**Figure 1:**
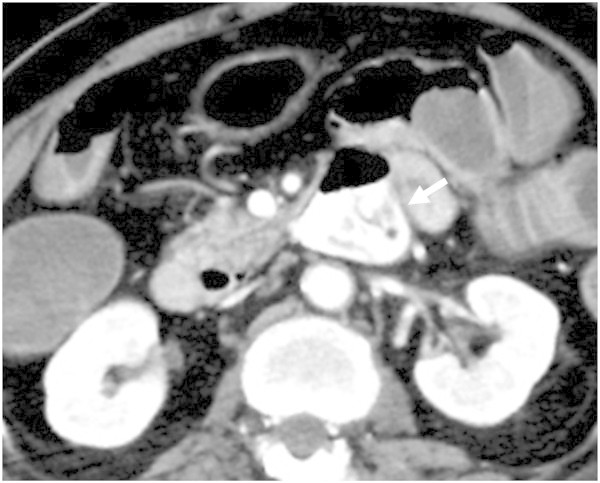
**CT angiography via the venous approach depicts extravasation (arrow) of contrast medium in a large diverticulum in the ascending duodenum.**

CT angiography via the venous approach depicted extravasation of contrast medium from a large diverticulum at the ascending duodenum (Figure 1). It was difficult to follow the aorta continuously to the feeding artery because the similarity in the CT values of the feeding artery and duodenum wall prevents their differentiation in our case. Control by gastro-endoscopy was attempted but failed because of massive bleeding. A gastric tube was inserted and transcatheter arterial embolization (TAE) was scheduled. TAE was performed at an angio–multidetector row CT (MDCT) facility (INFX 8000-C Aquilion CX, Toshiba Medical, Tokyo, Japan) that utilizes a common tabletop that enables angiography or MDCT to be obtained without transferring the patient.

**Figure 2:**
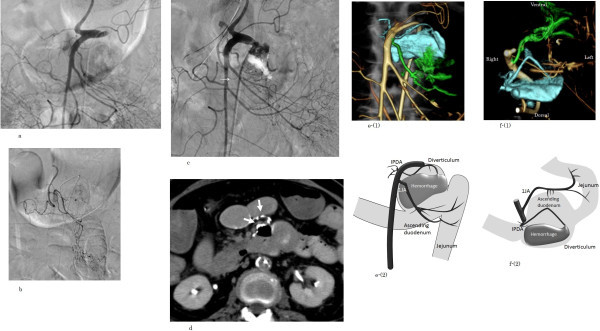
**The diagnostic approach for exploring the responsible artery for hemorrhage.** **a:** Angiography of the common tract of the superior mesenteric artery (SMA) and splenic artery does not depict extravasation of contrast medium. **b:** Selective arteriography of the first jejunum artery depicts the suspicious extravasation (*) of contrast medium which was difficult to differentiate from the duodenal wall. **c:** Superior mesenteric arteriography immediately after embolization depicts the patent first jejunum artery trunk (arrow) with partial occlusion and the movement of the duodenum containing air prevented to interpret the existence of extravasation of contrast medium. **d:** CT following transcatheter arterial embolization with n-butyl cyanoacrylate lipiodol (NBCA-Lp) of the first jejunal branch artery reveals accumulation of NBCA-Lp at the ventral wall of the ascending duodenum and the jejunum (arrows). **e (1):** A volume-rendered (VR) image obtained during superior mesenteric arteriography (SMA) (anterior–posterior view). **e (2):** Diagram of e (1) shows the extravasation and relevant arteries of the first jejunal branch artery (1JA, green) and inferior pancreatic duodenal artery (IPDA, blue). **f (1):** A VR image obtained during SMA (caudal–cranial view). **f (2):** Diagram of f (1) shows supply of the ventral and dorsal walls of the duodenum by 1JA and IPDA, respectively.

Selective arteriography of the superior mesenteric artery (Figure 2a), celiac artery, gastroduodenal artery, and dorsal pancreatic artery using a 4 F hook-shaped catheter (Cook Medical, Bloomington, IN) and a microcatheter (2.4 F, straight Sirabe, PIOLAX, Kanagawa, Japan) revealed no extravasation of contrast medium probably because of hypotension, or intermittent bleeding. MDCT was then performed during superior mesenteric arteriography (MDCT-SMA) using an MDCT scanner with 64 detector rows (Aquilion 64, Toshiba; 0.5 sec/rotation; reconstruction slice thickness/interval, 0.5 mm/0.5 mm) during administration of 30 ml of non-ionic contrast medium (175 mg of iodine per ml) through the hook-shaped catheter at a rate of 3 ml/sec using an automatic injector (Mark V Provis, Medrad, Pittsburgh, PA). MDCT-SMA depicted accumulation of contrast medium in the diverticulum and then, the hemorrhage-responsible-arteriography volume rendered (HRA-VR) image was created. Because of the time required to process the HRA-VR image and because the patient was in a state of hemorrhagic shock and receiving continuous blood transfusion, at this point the emergency surgeon asked us to attempt hemostasis by TAE of the suspicious artery. The 1JA was catheterized easily but catheterization of the IPDA was difficult. Selective 1JA angiography depicted suspicious extravasation of contrast medium in the diverticulum (Figure 2b). Although the extravasation did not indicate a distinct contrast medium leakage and difficult to differentiate from the jejunum wall, we determined to conduct embolization because of the life-threatening situation. N-butyl cyanoacrylate (NBCA, B. Braun, Melsungen, Germany) was prepared as a liquid embolic material by mixing 0.1 ml of NBCA with 0.7 ml of lipiodol (Lp) using a 1 ml syringe. Before embolization, the microcatheter was flushed with 5% glucose solution to prevent polymerization of NBCA in the microcatheter. TAE was conducted with 0.3 ml of the NBCA–Lp mixture. Superior mesenteric arteriography did not depict the extravasation of the contrast medium (Figure 2c), and the patient’s blood pressure temporarily rose to 110 mmHg. However, repeat MDCT without contrast medium depicted accumulation of NBCA-Lp at the ventral wall of the duodenum but no accumulation of NBCA-Lp in the diverticulum (Figure 2d). Soon after, the patient’s blood pressure dropped again to the 50 mmHg level.

At the moment that TAE of 1JA was completed, the HRA-VR image was displayed on the angiography monitor. The image demonstrated that the ventral and dorsal walls of the ascending duodenum were supplied by 1JA and IPDA, respectively, (Figure 2e (1), 2e (2)) and also that the large hemorrhagic diverticulum situated at the cranio-dorsal site was mainly supplied by the IPDA branching directly from the right wall of the SMA. Abrupt angle branching of the IPDA from SMA was also revealed (Figure 2f (1), 2f (2)).

**Figure 3:**
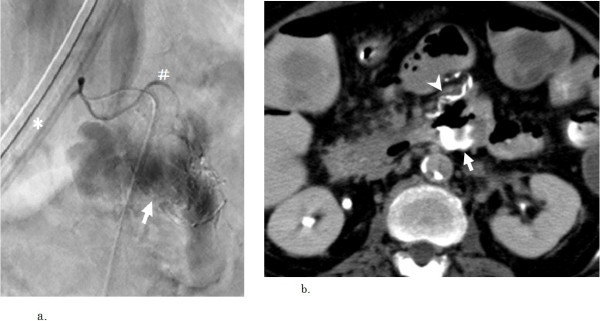
**The final angiography and CT after embolization.** **a:** Selective inferior pancreatic duodenal arteriography depicts extravasation of contrast medium (arrow) and reveals the dorsal duodenal branch artery as the responsible artery (#). (* gastric tube). **b:** CT immediately after transcatheter arterial embolization with n-butyl cyanoacrylate lipiodol (NBCA-Lp) depicts NBCA-Lp accumulation corresponding to the ventral duodenal wall (arrowhead) and the diverticulum (arrow) at the ascending duodenum.

It was possible to insert a manually angled microguidewire (0.014 inch, Hi-Lex, Hyogo, Japan) to the inlet of the IPDA but the abrupt branching angle of the artery prevented advance of the straight microcatheter across the guidewire. A commercially available angled-tip microcatheter (1 cm, 45 degree bending, 2.4 F, double-angle Sirabe, PIOLAX) was then inserted. Selective angiography of the IPDA via the microcatheter depicted a duodenal branch artery to the dorsal wall of the ascending duodenum as the responsible artery, and massive extravasation of contrast medium (Figure 3a). TAE of the responsible artery was conducted with the NBCA-Lp mixture, which was slowly injected through the microcatheter under fluoroscopic control until the duodenal dorsal branch artery was visualized. TAE was completed with 0.5 ml of NBCA-Lp mixture.

Angiography of the IPDA and plain CT immediately after the procedure depicted occlusion of the responsible artery at the duodenal wall and accumulation of NBCA-Lp in the diverticulum, respectively (Figure 3b). The patient’s blood pressure rose to 130 mmHg and was maintained at this level. Total fluoroscopic time was 2584 seconds. The anemia resolved the following day and no further blood transfusion was required. Gastroendoscopy one week after the procedure depicted a duodenal hemi-circumferential ulcer at the ventral wall of the ascending duodenum. The patient was prescribed anti-ulcer medication and was discharged at 14 days. Follow-up gastroendoscopy at 1 month revealed no residual ulcer. No symptoms related to hemorrhage were found at the last follow-up at 12 months.

## Discussion

Of the eight previously reported cases of hemorrhagic duodenal diverticulum treated with TAE (Kwon et al. 2009), seven were located in the descending duodenum and one in the transverse duodenum. Treatment of hemorrhagic diverticulum in the ascending duodenum by TAE is yet to be documented.

Based on their study of 164 cadavers, Murakami et al. (Murakami et al. 1999) documented the following branching patterns of the pancreaticoduodenal artery: IPDA arising from the common trunk with the upper jejunal artery (55.6%); IPDA arising directly from the SMA (24.2%)(the pattern described as typical in many textbooks); anterior IPDA (AIPDA) arising from the common trunk with the upper jejunal artery (11.3%); and AIPDA and posterior IPDA (PIPDA) arising independently from the SMA (3.3%). They reported that when the common tract of the IPDA and jejunal artery branched from the SMA, the ascending duodenum was supplied mainly by the jejunal artery; when the IPDA branched directly from the SMA, the ascending duodenum was supplied mainly by the IPDA; and when the AIPDA and PIPDA branched independently from the SMA, the ventral wall of the ascending duodenum was supplied by the upper jejunal artery while the dorsal wall was supplied by the PIPDA (Murakami et al. 1999). In the present case, the IPDA branched directly from the SMA, and the ventral and dorsal walls of the ascending duodenum were supplied from the 1JA and the IPDA, respectively, indicating anatomical rarity supplying the ascending duodenum.

Because the hemorrhagic diverticulum was situated at the cranio-dorsal wall of the ascending duodenum and because TAE of the suspected responsible artery from the 1JA was unable to achieve hemostasis, we found that the dorsal duodenal artery from the IPDA might be the artery responsible for the hemorrhage. Although arterial bleeding from the ascending duodenum is encountered rarely, in this situation it is imperative to consider the possible relevance of the 1JA and/or the IPDA. In the present case, irrespective rarity of the anatomical branching, the HRA-VR image was useful for depicting the responsible artery and the abrupt angle of its branching from the SMA, enabling successful catheterization of the IPDA with a pre-angled microcatheter. The HRA-VR image was useful to create the catheter treatment strategy in our case.

Possible adverse events following TAE for hemorrhagic duodenal diverticulum include ischemic damage, duodenal obstruction, pancreatitis, and re-bleeding. In an experimental swine study, embolization of three or fewer vasa recta with NBCA-Lp induced no damage or necrosis of the mucosal or submucosal layers in one-fourth of the intestinal circumference, while embolization of five or more vasa recta induced total necrosis of the whole intestinal circumference (Jae et al. 2008; Ikoma et al. 2010). The number of embolized vasa recta in the experimental study does not always assure the safety of embolization with NBCA-Lp in human. Kwon reported a case of duodenal obstruction caused by duodenal wall thickening and periventricular fibrosis that occurred 2 weeks after TAE had been conducted for duodenal diverticular hemorrhage (Kwon et al. 2009). In the present case, several vasa recta were occluded and a hemi-circumferential ulcer detected on the ventral wall of the ascending duodenum after TAE, was cured by conservative medicine. It took several minutes for HRA-VR image to come out on the monitor of angiography room, If HRA-VR derived from MDCT during aortography created more speedily, the embolization of the first jejnum artery would be avoided.

In conclusion, although hemorrhagic diverticulum in the ascending duodenum was supplied by the IPDA, the HRA-VR depicted the artery responsible for hemorrhage, enabling to create the catheter treatment strategy and leading to the successful treatment by NBCA-Lp embolization.
